# Chromosome level genome assembly of the American sloughgras*s (Beckmannia syzigachne)*

**DOI:** 10.1038/s41597-026-06836-w

**Published:** 2026-04-16

**Authors:** Wei Tang, Chuanlin Yin, Huangjie Gao, Zhongxian Lu, Yongliang Lu, Hongxing Xu

**Affiliations:** 1 State Key Laboratory of Rice Biology and Breeding, China National Rice Research Institute, Hangzhou, 311401 China; 2 College of Life Sciences, China Jiliang University, Hangzhou, 310018 China; 3 State Key Laboratory for Managing Biotic and Chemical Threats to the Quality and Safety of Agro-products, Institute of Plant Protection and Microbiology, Zhejiang Academy of Agricultural Sciences, Hangzhou, 310021 China

**Keywords:** Plant genetics, Genome

## Abstract

American sloughgrass [*Beckmannia syzigachne* (Steud.) Fernald] is a problematic annual grass weed in winter wheat fields of China, which causes great loss of wheat yield. A lack of high-quality genome resources has hindered understanding of the Herbicide resistance characteristics and ecological adaptations. Here, we combined Illumina short read, PacBio long-read, and high-throughput chromosome conformation capture (Hi-C) sequencing technologies to generate a high-quality, chromosome-scale genome assembly of *B. syzigachne*. The genome assembly was 3.19 Gb in size, consisting of seven pseudo-chromosomes. The contig and scaffold N50 values were 62.2 Mb and 431.7 Mb, respectively. The genome assembly completeness was estimated at 97.1% by BUSCO assessment. Annotation revealed 36,944 protein-coding genes and 88.83% repeat sequences. This high-quality genome assembly is a valuable resource for future fundamental research and agricultural management of *B. syzigachne*, and provides significant new insights into the herbicide resistance as well as the adaptive evolution of *B. syzigachne*.

## Background & Summary

*Beckmannia syzigachne* (Steud.) Fernald, commonly known as American sloughgrass, is an annual grass weed characterized by its scabrous leaves and spikelets, and is indigenous to Asia and North America. It is one of the most significant weeds in the wheat fields of the lower-middle reaches of the Yangtze River. This weed thrives particularly well in moist soil and has become a major problem in wheat (*Triticum aestivum* L.) or rapeseed (*Brassica napus* L.) fields that are rotated with rice (*Oryza sativa* L.) in the Yangtze River Valley and the southwestern region^1^. Due to its rapid growth, competitive ability, and capacity for rapid multiplication, this weed can reduce wheat yield by up to 50% or even cause crop failure if left uncontrolled^2,3^. Currently, chemical control is the primary strategy for managing *B. syzigachne*. Herbicides such as fenoxaprop-P-ethyl, clodinafop-propargyl, sethoxydim, and pinoxaden have been used post-emergence to control *B. syzigachne*. Moreover, *B. syzigachne* has evolved resistance to three known herbicide sites of action in China and Japan^4,5^. However, the widespread use of these herbicides has led to the development of resistance in *B. syzigachne*, and the current understanding of the molecular basis of *B. syzigachne* herbicide resistance is limited. Therefore, elucidating the mechanisms underlying herbicide resistance in *B. syzigachne* is crucial for effective weed management.

Genomic analysis is a powerful approach for identifying herbicide resistance mechanisms and adaptive alleles, enabling the discovery of target-site mutations, detoxification-related gene families, and regulatory elements associated with resistance^6–10^. For instance, genomic resources have facilitated the identification of ALS and ACCase target-site resistance mutations in Lolium rigidum and Echinochloa crus-galli, informing the design of molecular diagnostics and resistance monitoring protocols^11^. Such insights lay the foundation for developing more targeted and sustainable weed management strategies. The lack of a high-quality *B. syzigachne* genome assembly has impeded a deeper understanding of this notorious weed. To address this issue, we generated a high-quality chromosome-scale *B. syzigachne* genome assembly using a combination of Illumina short reads, PacBio high-fidelity (HiFi) reads, and high-throughput chromosome conformation capture (Hi-C) data. This resource is expected to provide a solid foundation for future development of novel or improved strategies for the efficient management of this economically devastating weed.

## Methods

### Sample collection and genomic DNA extraction

Seeds of *Beckmannia syzigachne* were collected in July 2021 from a wheat field in Yangzhou, Jiangsu Province, China (32.39°N, 119.42°E), where the species is known to exhibit resistance to ACCase-inhibiting herbicides. Seeds of American sloughgrass were collected in field and grow in the laboratory. After germination, seedlings of Beckmannia syzigachne were transferred to plastic pots filled with a 3:1 mixture of peat and vermiculite and grown in a controlled growth chamber under the following conditions: 16-hour light / 8-hour dark photoperiod, daytime temperature of 25 °C and nighttime temperature of 20 °C, and relative humidity maintained at 60–70%. Plants were watered regularly with distilled water and grown without exposure to herbicides or chemical treatments. Young leaves were collected for DNA isolation and whole-genome sequencing. The leaves were collected for RNA-sequencing (RNA-seq) and transcriptome assembly. The samples were immediately flash-frozen in liquid nitrogen after harvest, and stored at −80 °C for subsequent nucleic acid extraction. DNA was extracted from fresh plants with a plant DNA extract kit (GK1051, Generay Biotech Co., Ltd., Shanghai, China).

### Illumina sequencing and genome survey analysis

**Fig. 1:**
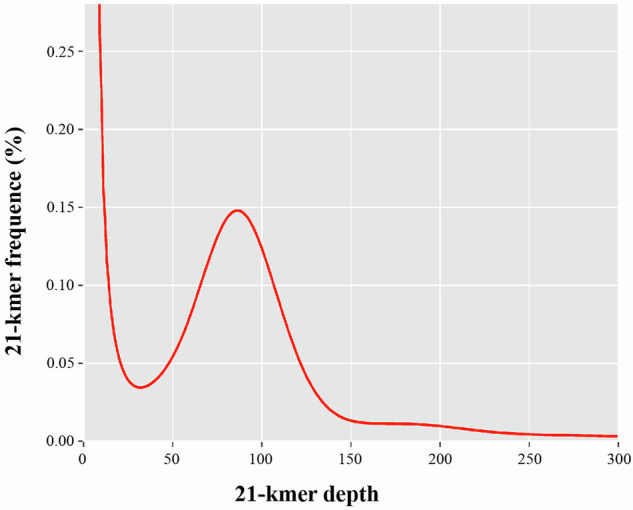
The 21-mer frequency distribution in *Beckmannia syzigachne* genome. (The x-axis is the k-mer depth, and y-axis represents the frequency of the k-mer for a given coverage).

Pair-end genome sequencing with a 150 bp insert size was performed using the Illumina TruSeq® Nano DNA library preparation kit (Illumina, San Diego, CA, USA), and the libraries were sequenced on an Illumina NovaSeq 6000 platform. The generated sequencing data were primarily processed using the NGSQC Toolkit^12^ (v2.3.3). This processing involved discarding reads that had adaptor contamination, reads with more than 5% unknown nucleotides (N), and paired reads with over 50% of bases having a quality score of less than 19 in either read. The filtering process yielded 295.05 GB of clean data (Table 1). Subsequently, a genome survey was conducted using Jellyfish^13^ (v2.2.717) with a k-mer setting of 21. GenomeScope^14^ (v2.0) was employed to estimate genome heterozygosity, repeat content, and size based on a k-mer-based statistical approach. The estimated genome size was determined to be 2.821 Gb, with a heterozygosity rate of 0.05% and a repeat sequence proportion of 74.33%. Additionally, the estimated GC content was 46.91% (Fig. 1, Table 2).

**Table 1:** Statistics of the sequencing data.

Library type	Platform	Data size (Gb)	Depth* (X)	Average length (bp)
WGS short reads	Illumina HiSeq X-Ten	315.09	~95.2	150
WGS long reads	Pacbio Sequel II	1,436.64	~478.6	14,745
Hi-C	Illumina HiSeq X-Ten	144.40	~48.1	150
RNA-Seq	Illumina HiSeq X-Ten	27.15	—	150

*For the convenience of calculation, the genome size of the *Beckmannia syzigachne* is set to 3 Gb.

**Table 2:** Statistics of k-mer analysis of *Beckmannia syzigachne* genome.

K-mer number	K-mer depth	Genome size (bp)	Repeat (%)	GC content (%)	Heterozygous ratio (%)
242,626,230,055	86	2,821,235,233	74.33	46.91	0.05

### Pacbio HIFI sequencing and draft genome assembly

HiFi sequencing libraries were prepared® using the SMRTbell™ Express Template Prep Kit 2.0. Shotgun genomic DNA sequence data were collected on the Pacific Biosciences Sequel II system using HiFi sequencing protocols and the Sequencing kit V2 (PN: 101-820-200). Sequence data collection was standardized to 30 hours for this study to allow ample time for multiple pass sequencing around SMRTbell template molecules of 10–25 kb, which yields high quality circular consensus sequencing (HiFi) results^15^. Raw base-called data were transferred from the sequencing instrument and imported into SMRTLink(v 8.0.0.80529) to generate HiFi reads using the CCS algorithm, which processed the raw data and generated HiFi FASTQ files with the following settings: minimum pass 3, minimum predicted RQ 20. After removing low-quality reads, a total of 1.43 Tb of PacBio polymerase clean data, comprising 97.54 million reads, was obtained, from which 84.61 Gb of high-quality CCS (HiFi) reads were generated for genome assembly. The N50 reads length was 16,124 bp, and the average read length was 14.7 Kb (Table 1). Then, the hifiasm^16^ (v0.16.1-r375) software with default parameters was used to assemble the PacBio HiFi reads into contigs, leveraging the high accuracy of HiFi reads and the string graph-based algorithm implemented in the assembler. Ultimately, contigs were generated based on the overlap graph. As a result, the draft genome of *B. syzigachne* was assembled with a total length of 3.19 Gb, composed of 4,308 contigs and the contig N50 was 64.24 Mb (Table 3).

**Table 3:** The statistics of different genome assembly.

Items	Contig length (bp)	Contig Numbers	Scaffold length	Scaffold Numbers
Total	3,191,659,073	4,308	3,191,685,773	4,041
Max	288,620,611	—	512,076,845	—
N50	64,242,125	11	431,717,085	4
N60	37,087,836	17	387,335,436	5
N70	21,800,903	28	356,547,123	6
N80	11,037,480	48	356,547,123	6
N90	1,040,752	140	320,682,383	7

### Hi-C sequencing and chromosome-scale assembly

**Fig. 2:**
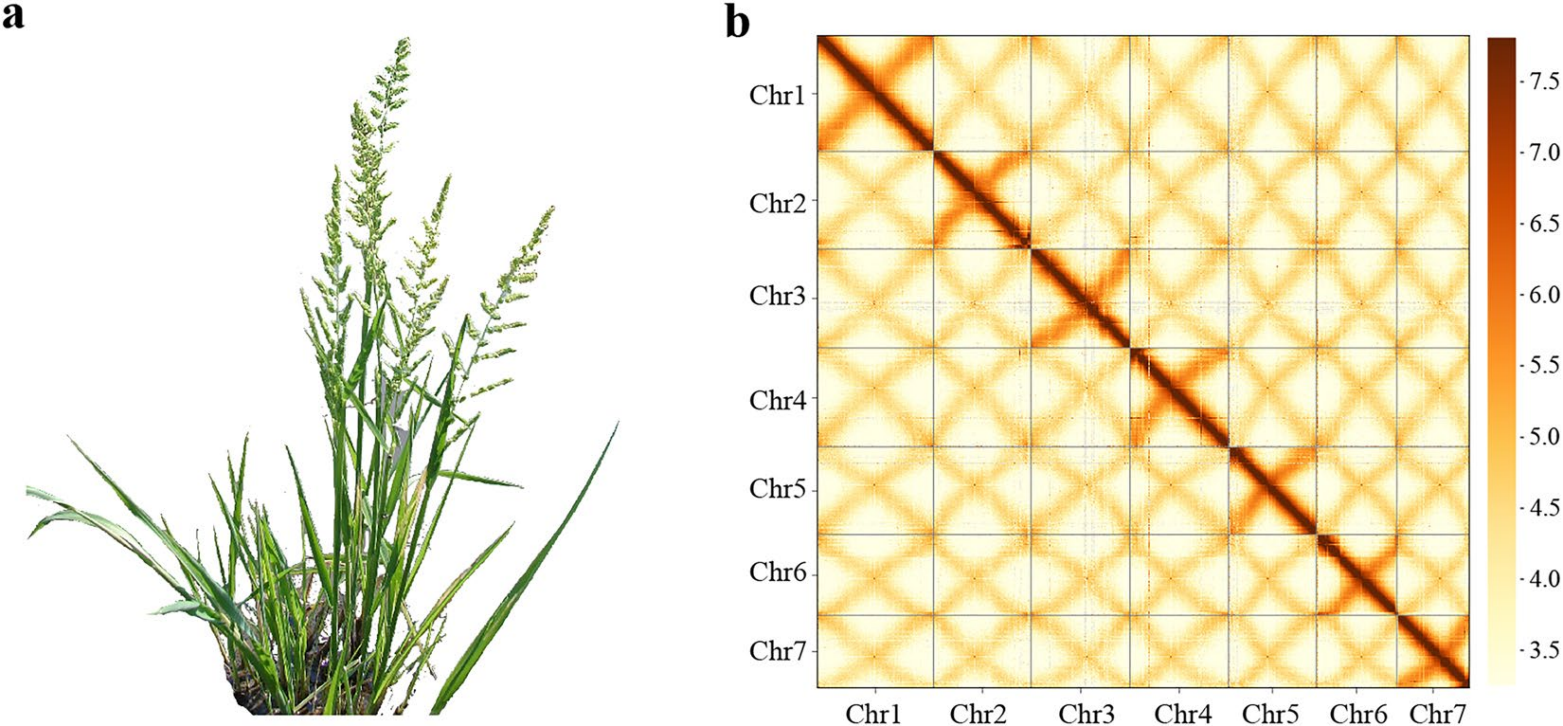
Hi-C interaction heatmap of *B. syzigachne* genome assembly.

For genome scaffolding, fresh leaves were collected and used to construct Hi-C libraries following a standard proximity ligation protocol^17^. Briefly, chromatin was crosslinked with formaldehyde, digested using a restriction enzyme, end-repaired and biotin-labelled, followed by proximity ligation, DNA purification, fragmentation, and enrichment of ligation junctions to generate the Hi-C sequencing libraries. The prepared libraries were sequenced on the Illumina NovaSeq 6000 platform (Illumina, USA) using a 150-bp paired-end strategy. After filtering the raw data to remove adapter sequences, low-quality bases, and reads with excessive ambiguous nucleotides by fastp^18^ ((v0.20.0)) with default parameters, 137.04 Gb of clean data were generated (Table 1). Valid Hi-C interaction pairs were used to scaffold the assembled contigs into seven pseudo-chromosomes using the LACHESIS^19^ pipeline (v201701), with parameters set as follows: CLUSTER_MIN_RE_SITES = 100, CLUSTER_MAX_LINK_DENSITY = 2, CLUSTER_NONINFORMATIVE_RATIO = 1, ORDER_MIN_N_RES_IN_TRUNK = 50, and ORDER_MIN_N_RES_IN_SHREDS = 50. Ultimately, 90.26% of the draft genome sequences (3.19 Gb) were anchored to 7 pseudo-chromosomes of *B. syzigachne* (Table 4). The pseudo-chromosomes were named based on scaffold size, from the largest to the smallest. Seven pseudo-chromosomes were assembled based on Hi-C interaction data, corresponding to the seven most strongly supported chromosomal clusters identified by LACHESIS. This is consistent with cytogenetic studies reporting that B. syzigachne has a diploid chromosome number of 2n = 14^20^. The final chromosome-scale assembly composed of 4,041 scaffolds with a scaffold N50 of 431.72 Mb (Table 3, Fig. 2).

**Table 4:** The statistics of pseudo-chromosomes.

Pseudomolecule	Contig Num	Length
chr1	42	512,076,845
chr2	80	431,717,085
chr3	44	436,825,958
chr4	29	435,626,328
chr5	45	387,335,436
chr6	23	356,547,123
chr7	11	320,682,383
Total anchored	274	2,880,811,158
Unanchored	4,034	310,874,615

### Transcriptome sequencing

For RNA extraction, four tissue samples from various parts of the *B. syzigachne* plant, including root, stem, leaf, ear, were collected. For ear tissues, samples from four different stages of ear were collected. RNA was extracted from fresh plants with a plant total RNA extract kit (Generay Biotech Co., Ltd., Shanghai, China). The transcriptome sequencing libraries from different tissues (root, stem, leaf, flower) were prepared using the Illumina True-seq transcriptome kit (Illumina, CA). The libraries were then sequenced on Illumina NovaSeq 6000 platform to generate 150 bp paired-end reads. Raw data (raw reads) of FASTQ format were first processed through fastp^18^ (v0.20.0) with default parameters. In this step, clean data (clean reads) were obtained by removing reads containing adapter, reads containing ploy-N and low-quality reads from raw data. Simultaneously, Q20, Q30 and GC content of the clean data were calculated (Table 1).

### Genome annotation (TEs, Genes, ncRNAs)

We applied a combined strategy that utilized both de novo search and homology alignment to identify the repeats. Based on the repetitive sequence database RepBase^21^. The raw transposable element (TE) library included all repeat sequences that were longer than 100 bp and had less than 5% “N” gaps. To obtain a nonredundant library, a combined of Repbase and the raw TE library processing was conducted using uclust^22^. Finally, RepeatMasker^23^ v.4.1.0 was employed for the repeat identification using the nonredundant library. The homology-based approach utilized RepeatMasker^24^ (v.4.1.0) and the Repbase library to identify known transposable elements (TEs). These identified TEs were subsequently aligned with the genome sequences using a TE protein database, RepeatProteinMask (v.4.1.032). Tandem repeats were predicted using Tandem Repeats Finder^25^ (v.4.0933). In the genome assembly, 83.26% repeat sequences were identified, among which 9.46% were DNA transposons and 67.22% were long terminal repeat retrotransposons (Table 5).

**Table 5:** Repetitive DNA composition of the *B. syzigachne* genome.

Type	Superfamily	Copy Number	Length	Percent (%)
Class II			295,558,476	9.26
	Tc1-Mariner	112,817	30,701,401	0.96
	Mutator	0	0	0
	CACTA	2,881	3,015,233	0.09
	PIF-Harbinger	4	529	0
	hAT	132,056	38,977,691	1.22
	Merlin	0	0	0
	Transib	0	0	0
	PiggyBac	0	0	0
	Crypton	0	0	0
	Helitron	3,949	651,811	0.02
	Maverick	0	0	0
	Others	588,673	228,990,613	7.17
Class I			2,166,329,022	67.87
	LTR/Gypsy	1,045,652	1,278,962,137	40.07
	LTR/Copia	148,793	106,111,056	3.32
	LTR/DIRS	2	121	0
	LTR/PLE	0	0	0
	LTR/unknown	618,085	760,679,805	23.83
	LINE	249,366	124,466,187	3.9
	SINE	378	46,265	0
	Others	3,755	1,348,745	0.04
Simple_repeat		0	0	0
Others		256,369	222,407,082	6.97
Unknown		94,514	39,444,060	1.24
Total		3,257,294	2,657,460,845	83.26

After masking repeat sequences, protein-coding and non-coding RNA (ncRNA) genes were classified using a combination of transcriptomic, homology searching, and *ab initio* prediction-based approaches. Utilizing Geta (https://github.com/chenlianfu/geta, v2.4.11) for gene structure prediction, an integrated, non-redundant, and complete gene set is obtained. This approach predicted 36,944 genes with an average gene length of 3,532.46 bp (Table 6). Functional annotation was performed for the predicted protein-coding genes via comparing with public databases including NCBI NR^26^/NT, EggNOG^27^, Pfam^28^, GO^29^, KEGG^30^, and UniProt^31^. Protein sequences were aligned to NCBI NR/NT, Uniprot by BLASTP v.2.10.1 (E-value ≤ 1e-5). EggNOG, Pfam, GO,and KEGG annotations were performed with eggNOG-mapper^32^ v2. Finally, 35,056 genes were functionally annotated in at least one of the above databases, accounting for 94.89% of the predicted protein-coding genes (Table 7).

**Table 6:** Summary of gene annotation of *B. syzigachne* genome.

Features	*B. syzigachne* genome
Number of proteins	36,944
Average gene length(bp)	3,532.46
Average cds length(bp)	1,272.06
Average exons per gene	4.98
Average exon length(bp)	315.3
Average intron length(bp)	493.29
Complete BUSCOs (%)	96.3
Complete and single-copy BUSCOs (%)	84.4
Complete and duplicated BUSCOs (%)	11.9
Fragmented BUSCOs (%)	1.0
Missing BUSCOs (%)	2.7

**Table 7:** Statistics of gene function annotation of the *Beckmannia syzigachne*.

Database	Count	Percentage (%)
BLASTP	27,280	73.84
BLASTX	26,759	72.43
GO	27,590	74.68
KO	9,765	26.43
Map	6,133	16.60
NR	34,869	94.38
NT	30,491	82.53
PFAM	28,633	77.50
eggNOG	23,445	63.46
Total_anno	35,056	94.89
Total_unigene	36,944	100

The prediction of the non-coding RNA gene set (ncRNA) was carried out across the genome. Initially, the data was aligned with the noncoding database of Rfam^33^ library v.11.0, for the annotation of genes encoding various non-coding RNAs including small nuclei RNAs (snRNAs), ribosomal RNAs (rRNAs), and microRNAs (miRNAs). The transfer RNA (tRNA) sequences were subsequently identified using tRNAscan-SE^34^ (v.2.0) with default parameters. These analyses yielded a total of 4,966 small nucleolar RNA (snoRNA) genes, 11,195 tRNA genes, 10,588 rRNA genes, and 2,792 microRNA (miRNA) genes (Table 8).

**Table 8:** Non-coding RNA statistics of the *Beckmannia syzigachne* genome.

Class	Type	Copy	Average length(bp)	Total length(bp)	%of genome
miRNA	miRNA	2,792	119.75	334,332	0.01048
tRNA	tRNA	11,195	75.26	842,552	0.0264
rRNA	18S	913	1723.10	1,573,189	0.04929
	28S	3,480	144.09	501,420	0.01571
	5.8S	872	158.20	137,949	0.00432
	5S	5,323	118.36	630,052	0.01974
snRNA	CD-box	4,704	105.42	495,896	0.01554
	HACA-box	82	126.61	10,382	0.00033
	splicing	180	148.88	26,798	0.00084
Total		29,541	154.11	4,552,570	0.14264

## Data Records

The Illumina, PacBio, and Hi-C sequencing data used for the genome assembly have been deposited in the NCBI Sequence Read Archive (SRA) under accession numbers SRR29499852^35^, SRR29692811^36^, and SRR29821161^37^ and BioProject accession number PRJNA1127069^38^. The transcriptome sequencing data used for genome annotation have also been submitted to the SRA database under accession number SRR29845012-SRR29845015^39–42^. The chromosomal assembly has been deposited to GenBank under accession number GCA_040954875.1^43^. The genome assembly and annotated genes have been deposited to the website repository (https://bioinformatics.cjlu.edu.cn/db/bsyzi).

## Technical Validation

Firstly, the final genome assembly of *B. syzigachne* was 3.19 Gb, which is notably larger than the genome size estimated from k-mer analysis (2.82 Gb). This difference of approximately 12% is likely attributable to the high proportion of repetitive sequences in the genome, which comprise around 88.8% of the assembly. Repetitive regions, including long transposable elements and tandem repeats, are prone to expansion during long-read assembly, whereas k-mer-based estimation can underestimate highly repetitive or complex regions. Therefore, the slightly larger assembly reflects both the biological complexity of the genome and the technical characteristics of long-read assembly, rather than an inconsistency in the data.

Secondly, the quality of the of *B. syzigachne* genome assembly was evaluated based on the contiguity, completeness, and correctness. Hi-C interaction analysis revealed clear interactions among the seven pseudo-chromosomes, and the interaction contact pattern was organized along the principal diagonal of the Hi-C heatmap (Fig. 1), supporting the accurate assignment and orientation of scaffolds at the chromosome level. Furthermore, 90.26% of the draft genome sequences were successfully ordered and oriented within the seven pseudo-chromosomes, resulting in a scaffold N50 of 431.72 Mb (Tables 3, 4). Approximately 300 Mb (~10%) of the genome could not be assigned to pseudo-chromosomes. These unplaced sequences primarily consist of highly repetitive regions and residual heterozygous contigs, including some small contigs (<100 kb), which are challenging to scaffold even with Hi-C data. Organelle-derived sequences were identified separately and excluded from the nuclear genome assembly. These results indicate that the assembly achieved a high degree of chromosome-scale organization and scaffold continuity, providing a robust framework for downstream analyses.

Genome completeness was evaluated using BUSCO^44^ (v5.2.2) with the poales_odb12 database. The genome assembly contained 97.1% complete BUSCOs (84.6% single-copy, 12.5% duplicated), with 1.5% fragmented and 1.5% missing genes. BUSCO analysis of the predicted gene set recovered 96.3% complete BUSCOs (84.4% single-copy, 11.9% duplicated), with 1.0% fragmented and 2.7% missing genes, indicating that the annotation captures the vast majority of conserved genes (Table 6). To further validate chromosome completeness, the canonical plant telomeric repeat motif (TTTAGGG) was detected at the ends of most pseudo-chromosomes with quartet^45^ (v1.2.5), confirming that the seven pseudo-chromosomes are largely complete and accurately assembled. Additionally, we evaluated the genome quality using Merqury^46^ (v1.3), yielding an average quality value (QV) of 41.84. Concerning correctness, all Illumina short reads were aligned to the genome assembly using BWA^47^ (v0.7.17), resulting in a high mapping rate of 98.94%. Overall, these findings indicate that the *B. syzigachne* assembly is characterized by high accuracy and completeness.

## Data Availability

The seeds used in this study are stored in long-term preservation at the State Key Laboratory of Rice Biology and Breeding, China National Rice Research Institute. All sequencing data and genome assemblies generated in this study are publicly available in established repositories. The Illumina, PacBio, and Hi-C sequencing data used for genome assembly have been deposited in the NCBI Sequence Read Archive (SRA) under accession numbers SRR2949985241, SRR2969281142, and SRR2982116143, respectively, associated with BioProject PRJNA1127069, while the transcriptome sequencing data used for genome annotation are available in the SRA under accession numbers SRR29845012–SRR2984501545. Additionally, the chromosomal assembly has been deposited in GenBank under accession number GCA_040954875.146, and the complete genome assembly along with the annotated genes can be accessed and downloaded from the dedicated website repository at https://bioinformatics.cjlu.edu.cn/db/bsyzi.

## Code availability

Programs used in data processing were executed with the default parameters except where otherwise specified in the Methods. No custom code was used in these analyses.
